# Expression profiling of some Acute Myeloid Leukemia - associated markers to assess their diagnostic / prognostic potential

**DOI:** 10.1590/1678-4685-GMB-2019-0268

**Published:** 2021-01-06

**Authors:** Nahla O. Mousa, Marwa Gado, Magda M. Assem, Kamal M. Dawood, Ahmed Osman

**Affiliations:** 1 Egypt-Japan University of Science and Technology (E-JUST), Basic and Applied Sciences Institute, Alexandria, Egypt.; 2 Cairo University, Faculty of Science, Department of Chemistry, Giza, Egypt.; 3 Cairo University, National Cancer Institute, Department of Clinical pathology, Giza, Egypt.; 4 Ain shams University, Faculty of Science, Department of Biochemistry, Cairo, Egypt.

**Keywords:** AML, BAX, ARC, PHF6, ASXL1

## Abstract

Investigating the etiological causes of acute myeloid leukemia (AML) at the molecular level should help in identifying targets and strategies that would increase the efficacy of the current management regimens. Some genes may act as molecular diagnostics, of these *ASXL1* and *PHF6* are involved in regulation of gene expression, and **BAX** , and *ARC*, are pro- and anti-apoptotic molecules, respectively. In this study, peripheral blood samples were collected from 54 recently diagnosed AML patients in addition to 20 healthy individuals (the control group). Cellular RNA was extracted from all the samples and were subjected to quantitative analysis of the transcript levels of the four selected markers. Our data showed a significant elevation in the expression levels of *PHF6* and *ARC* in AML patients, when compared to the controls (77.8% and 83.3%, respectively). On the other hand, *ASXL1* and *BAX* exhibited increase, to a lesser extent, in the expression levels of the AML patients (52% and 55.6%, respectively). Our study also showed that the expression levels of *ARC* and *PHF6* exhibited a concomitant increase and this could be correlated with poor prognosis of the cases. Thus, we can suggest these markers as reliable prognostic markers for prediction of AML outcomes.

## Introduction

Acute myeloid leukemia (AML) is one of the heterogeneous disorders originating from the neoplastic alteration of a hematopoietic stem cell, in which the transformed malignant cells exhibit minimal or aberrant differentiation. These transformed cells proliferate and primarily accumulate in the bone marrow and peripheral blood and may attack other organs/systems such as the liver, the lung and central nervous system. Common consequences include neutropenia, anemia, and thrombocytopenia may lead to death if untreated (Tallman *et al*., 2005). 

In normal scenario, the interplay between intracellular signaling systems results in exerting a precise balance/equilibrium between pro-apoptotic and anti-apoptotic mechanisms, depending on the exact cellular context. However, during the process of initiation and progression of AML as well as other malignancies, such balance is shifted in favor of anti-apoptotic and proliferative signals. 

In early stage myelodysplastic syndromes (MDS), CD34^+^ cells exhibited elevated expression levels of the pro-apoptotic proteins such as Bax and Bad, which indicate an overall apoptotic trend (Parker *et al*., 2000). On the other hand, the anti-apoptotic protein NOL3 (nucleolar protein 3; also known as apoptosis repressor with caspase recruitment domain; ARC), which plays protective roles in the heart (Li YZ *et al*., 2007; Li Y *et al*., 2009), skeletal muscles (Koseki *et al*., 1998) and the brain (Hong *et al*., 2003), acts through the downregulation of both extrinsic and intrinsic apoptotic pathways. (Gustafsson *et al*., 2004; Heikaus *et al*., 2008; Nam *et al*., 2004). In addition, NOL3 downregulates the expression of the tumor suppressor protein, p53. 

BAX or Bcl2-Associated X protein, a member of the Bcl2 protein family whose members positively or negatively regulate apoptotic processes, heterodimerize with Bcl2 and functions as an apoptotic activator, where it stimulates the opening of the mitochondrial voltage - dependent anion channel and thus leads to the loss of the membrane potential and the release of cytochrome C. Meanwhile, BAX expression is mediated by the tumor suppressor p53 and it is involved in its apoptotic activities (RefSeq, 2008).

Chromatin remodeling is one of the vital regulatory processes that is being targeted during malignancy. *ASXL1*; additional sex combs-like 1, encodes a chromatin binding protein and may act to enhance or repress gene transcription in localized areas by modifying chromatin structure (Katoh 2015)****.* ASXL1*** was found to be mutated in several human malignancies (Abdel-Wahab *et al*., 2012; Shih *et al*., 2012) as well as in MDS and in AML(Devillier *et al*., 2012).

Similarly, plant homeodomain finger 6 (*PHF6*), which is an X-linked gene, produces another protein containing four nuclear localization signals and two imperfect PHD zinc-finger domains (Lower *et al*., 2002). This protein is thought to play roles in transcriptional regulation and/or chromatin remodeling (Lower *et al*., 2002; Voss *et al*., 2007). PHF6 mutations were also found in 2-3% of adult AML and as is the case with other X-linked traits, inactivating mutations occur more frequently in males than females (Van Vlierberghe *et al*., 2011; Yoo *et al*., 2012).

However, all the aforementioned studies interrogated the above markers from the mutational analysis point of view only, and to our knowledge, none of these studies has addressed the expression levels as a possible mechanism contributing to the initiation and/or progression of AML. Therefore, in this study, the expression profile of these markers was investigated to complete the picture and to shed some light on the involved mechanisms in AML.

## Material and Methods

### Subjects

In this study, 54 acute myeloid leukemia patients diagnosed at the department of clinical pathology, National Cancer institute (NCI, Cairo University, Egypt). The research methodology complied with the institutional ethical regulation of the NCI (ethical approval #MS2001415019.4, the NCI Ethical Review Committee) and signed consent forms were also obtained from the patients. AML diagnosis/typing was performed according to the French-American-British (FAB) classification system. In addition, 20 healthy individuals enrolled and constitute a control group (Same age and sex as the patients group). Pretreatment peripheral blood was collected from subjects enrolled in the study. Bone marrow aspirates were also collected from all the patients. Samples were collected in K_2_-EDTA vacutainers and nucleated cellular fractions were processed for quantifying the gene expression levels of the selected markers to evaluate their diagnostic/prognostic potential. 

### Primers/probes design

PCR primers/probes were designed to achieve specific amplification of the target amplicons for the detection and validation of transcript abundance variation and to avoid non-specific amplification. Primers and probes were designed to have annealing temperature between 60± 2 °C with 45-55% GC content and product size ranged between 100 to 180 base pairs. Self- and hetero-dimerization and hairpin formation were also checked (OligoAnalyzer 3.1; Integrated DNA Technologies, Lowa, United States). To avoid amplification of genomic DNA contamination in RNA preps, the design took in consideration to have the probe or one of the primers spanning an exon-exon junction, or the set would anneal to regions on two different exons that are flanking a relatively long intron, hence amplification would occur of cDNA only by controlling the extension time. Alternatively, in cases where above considerations were hard to fulfill, RNA samples were treated with RNase-free DNaseI (Promega, Wisconsin, USA). The primers and probes used in this study (Eurofins, Luxembourg) are listed in Table1.

**Table 1 -:** List of primers and probes, showing expected product size of each set.

List of primers/probes		Product size (bp)
ASXL1 Forward	5’- ACTCGGATGCTCCAATGACACC -3’	130
ASXL1 Reverse	5’- AAAACAACCCCTCTCCTCCTCTT -3’	
ASXL1 (Texas Red/BHQ2)^*^	5’- GTCATAGAGGCAGAAGGACTAAAGG -3’	
PHF6 Forward	5’- GGACATACCACTACCACTGTGC -3’	103
PHF6 Reverse	5’- TTTAGGTGATGAGGGCTCCAGTT -3’	
PHF6 (Texas Red/BHQ2)^*^	5’- GAAAACTGCACATAACTCCGAAGC -3’	
BAX Forward	5’- GGTTGTCGCCCTTTTCTACTTTG -3’	127
BAX Reverse	5’- AGGAAGTCCAATGTCCAGCCCA -3’	
BAX (HEX/BHQ1)^*^	5’- TGCACCAAGGTGCCGGAACTGAT -3’	
ARC Forward	5’- AAAGGGACGAGTCCGAAGATTC -3’	115
ARC Reverse	5’- GGAGTTTATTCACTTCCAGCGGT -3’	
ARC (Cy5/BHQ2)^*^	5’- TGCTGGATAGGACCTGGGATGCT -3’	
ACTB Forward	5’- GCGAGAAGATGACCCAGATCA -3’	118
ACTB Reverse	5’- GAGTCCATCACGATGCCAGTGG -3’	
ACTB (FAM/TAMRA)^*^	5’- CTATCCAGGCTGTGCTATCCCTG -3’	

^*:^ Labeling dyes for the probes (reporter and quencher, respectively).

### Flow cytometric immunophenotyping

Immunophenotyping was performed on bone marrow aspirate or peripheral blood samples for the following antigens: MPO, CD4, CD7, CD11c, CD13, CD14, CD33, CD34, CD117, HLA-DR and Tdt.

Cytogenetic Studies

All cytogenetic analysis was performed at the Department of Clinical Pathology-National Cancer Institute according to standard protocols.

Sample processing and total RNA isolation

The nucleated cells were isolated from the whole blood samples and bone marrow aspirates after plasma separation followed by lysis of erythrocytes using RBC’s lysis buffer (Promega, Wisconsin, USA). Total RNA was extracted from isolated cells using 1ml TRIZOL (Thermo Fisher Scientific, Massachusetts, USA) following the manufacturer recommended instructions (Chomczynski and Sacchi, 1987). RNA was precipitated off the aqueous phase by equal volume of isopropanol with GeneElute (Sigma, Missouri, USA) as an inert carrier. The concentration and the quality of RNA samples were determined spectrophotometrically (A_260_ and A_280_) (Shimadzu, Kyoto Prefecture, Japan).

Quantitative RT-PCR analysis

cDNA synthesis was carried out using 1 μg total RNA in a 20 µl reaction volume using MMLV reverse transcriptase (Promega, Wisconsin, USA) following the vendor’s recommendations, in which target non-specific priming was achieved by using 0.5 µl of 100 µM oligo-dT and 0.5 µl of 100 µM random decamer (Eurofins, Luxembourg). Negative RT (NRT) control reactions were performed, at which the enzyme was omitted, to monitor non-specific amplification off genomic target sequences.

Quantitative PCR amplification was carried out using Mx3000P thermal cycler (Agilent Technologies, Inc., California, USA). The 20 µl-reactions contained cDNA samples or NRT reaction (equivalent to 5 ng total RNA), 10 µl 2X GoTaq probe qPCR or GoTaq qPCR master mixes (Promega, Wisconsin, USA), 0.25 µl of each of the gene-specific primers (10 µM) with or without 0.3 µl TaqMan probe (10 µM) for TaqMan or SYBR green assays, respectively. The volume was brought to 20 µl with nuclease free water and the reactions were run in triplicates for precision. Template-free control reactions (No-Template Control; NTC) were also run to detect any possible reagents’ contamination. Reactions were subjected to the following thermal profile: initial denaturation at 95 °C for 3 min, followed by 30 s, 60 °C for 30 s and 72 °C for 30 s. The program was terminated with dissociation curve analysis in case of SYBR green assay. Data was collected and analyzed using the MxPro Q-PCR software (Agilent Technologies, Inc., California, USA).

Relative quantification of gene expression

Gene expression levels of candidate genes in patients’ samples were determined as fold change relative to that of control group. Levels were calculated using the Livak method (Livak and Schmittgen, 2001). Data was normalized using β-actin as a normalizer gene. The Cut off value for the expression levels of the studied genes were calculated using ROC curve analysis and the values was set 90% sensitivity and 98% specificity. RQ (Relative Quantification) values > 1.5 was considered as over expression, RQ < 0.9 was considered as downregulation and RQ values between 0.9 and 1.5 was considered as no change in expression levels.

Statistical analysis

Data was analyzed using Statistical Package for Social Sciences (SPSS, V.20; developed by IBM, New York, USA). Data were expressed as mean ± standard deviation/median along with range and as frequency/percentage (for Numerical and qualitative data respectively). For comparing qualitative values, Chi-square test (Fisher’s exact test) was used and Student *t*-test or Mann-Whitney test was used for correlating quantitative variables. Kruskal-Wallis test was used for comparing more than two groups. Correlation was evaluated using Pearson product-moment. Kaplan-Meier analysis was used for survival investigation (XLSTAT software; v.2016.05.34579) which was built using survival data from the patients’ records from the Department of Cancer Epidemiology and Biostatistics, NCI, Cairo University, Egypt. A *P*-value < 0.05 was considered significant. 

## Results

### Validation of the efficacies of the in-house primers/probe design

In order to validate the efficacies of the in-house designed primers / probe combinations, the probes and/or primers of the tested genes were used in singleplex formats, using cDNA from control subjects as templates, in Taqman and SYBR-green assays. The preliminary data showed that tested primers/probe combinations resulted in the amplification of target sequences with typical sigmoidal amplification curves. In the meantime, NTC and negative RT (NRT) control reactions resulted in no detectable amplification. Furthermore, signal specificity, in case of SYBR-green I assay, was verified with the dissociation - curve analysis, where each amplicon exhibited a single, specific melting event. 

Clinical and molecular data

The current study included 54 patients and 20 healthy controls. The ages of patients and healthy controls ranged between 20-60 years with a mean age of 41.48 + 13.85 years and 35.45 + 15.08 years, respectively, with no significant differences between patients and healthy control groups in age or sex ratio.

Initial clinical data of the newly diagnosed AML patients at the time of diagnosis is shown in Table S1. The data shows a significant increase in the total leukocyte count in AML group (53.6±74.1 x 10^3^/mm^3^), as compared to that of the control group (5.4 ±1.5 x 10^3^/mm^3^). On the other hand, the hemoglobin levels were significantly decreased as compared to that of the control group (7.6 ±2.0, 12.8 ±1.4 g/dl, respectively; *P*<0.001). Similarly, platelets count also showed sharp reductions in AML patients as compared to that of the Control group (57.9±84.7, 249.9±68.3 x 10^3^/mm^3^, respectively; *P* < 0. 001). 

AML cytogenetic analysis and immunophenotyping

Bone marrow aspirates revealed that among the AML patients enrolled in this study, AML-M1 subtype was the most common AML subtype of the enrolled patients followed by M2 subtype with incidence rate of 35.2% and 18.5%, respectively. Regarding the cytogenetic analysis, it was found that 24% of the AML patients had chromosomal aberrations; *t*(15;17, 16.6%), t(8;21, 3.7%), t(9;22, 3.7%). 

The frequency of expression of 11 surface antigens in AML patients obtained through immunophenotyping analysis is represented in Table S2. 

### Mutation and gene expression analyses

FLT3 mutation

Assessment of FLT3 gene mutation status revealed that 16.7% of the patients had FLT3-internal tandem duplication (ITD) mutation, while the rest had wild-type FLT3.

The expression levels of the 4 selected genes were assessed using qRT-PCR assay.

ASXL1 expression

**Figure 1 -:**
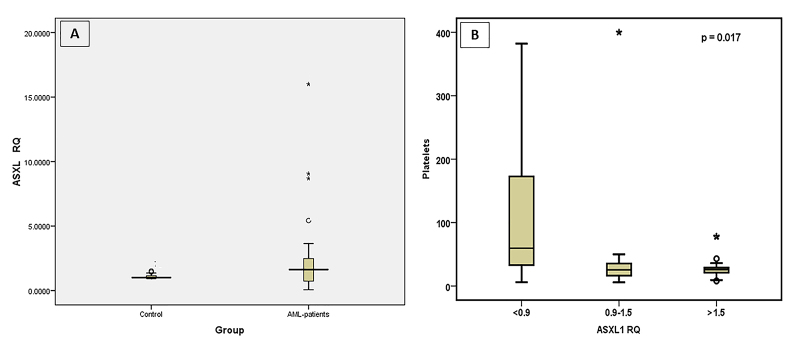
Significance of ASXL1 expression. ASXL1 expression levels, represented as RQ value, in AML patients and in Control groups (A). Correlation between platelets numbers and ASXL1 expression levels (B).

The expression levels of ASXL1 in AML patients was not significantly different from normal subjects (Figure 1A), however, transcript levels showed a significant increase in 28 patients (52%) with RQ range from 1.62 to 16, while it exhibited a reduction in 13 patients (24%) with a RQ range from 0.065 to 0.9 (Figure 1A and Figure 5). The mean expression level; relative quantity (RQ) in AML patients was 2.24 folds with a median of 1.62-fold.

ASXL1 expression was significantly correlated with platelets count (*P* =0.017) (Figure 1B) but no significant correlation was found with sex (*P* = 0.946), age (*P* =0.092), hemoglobin concentration (*P* =0.805), TLC (*P* =0.246) or even leukemic blasts count (*P* =0.933). Also, no correlation was found between AML FAB subtypes, cytogenetic analysis (*P* =0.697) or FLT3 mutation (*P* =0.431) with the expression of ASXL1. However, ASXL1 expression showed a strong association with the expression of CD4 surface markers in myeloid cells in leukemia patients (CD4 negative cells had lower ASXL1 expression levels (*P* = 0.022).

PHF6 expression

**Figure 2 -:**
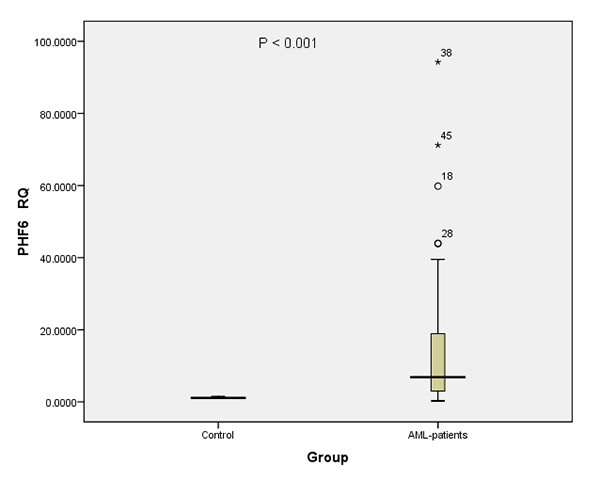
Significance of PHF6 expression. PHF6 expression levels, represented as RQ value, in AML patients and in Control groups.

PHF6 expression was significantly different between AML patients and control subjects. The majority of AML patients (77.8%) exhibited significant increase in the transcript level of this marker (*P* <0.001) compared to normal population with a RQ range from 1.7 to 94.2, as compared to that of the control group, with a mean value of 13.91 folds (Figure 2 and Figure 5) and a median value of 6.62 folds. PHF6 expression was found to be positively correlated with the percentage of blast cells in bone marrow aspirates (*P*=0.046). On the other hand, there was no significant correlation with any of the other parameters. However, PHF6 data showed a trend of association of elevated expression levels of this marker in patients with CD34+ surface marker (*P* =0.063). Moreover, when PHF6 expression levels data were analyzed in different AML subtypes, it could be clearly observed that AML-M0 subtype could be exhibited the highest expression rates with a median value of 14.96 folds (data not shown).

ARC expression

**Figure 3 -:**
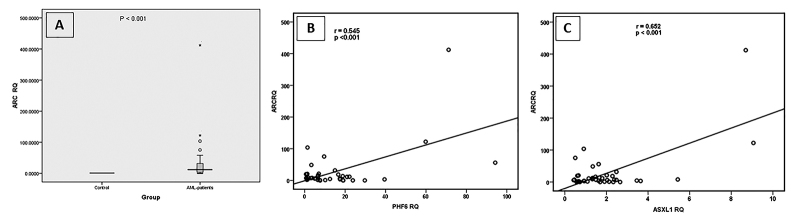
Significance of ARC expression. ARC expression levels, represented as RQ value, in AML patients and in Control groups (A). Correlation between ARC expression and PHF6 expression (B). Correlation between ARC expression and ASXL1 expression (C).

ARC expression in the AML patients was significantly different from the control subjects. Compared to the normal control group, AML patients showed an increase in ARC expression rates, where 83.3% of the patients exhibited a statistically significant increase (*P* =0.002) with a RQ range from 1.59 to 412.14 and mean value of 32.4 and a median RQ value of all AML patients of 7.41 (Figure 3A and Figure 5). In addition, a slight correlation was found between sex and the expression rate of ARC (*P* =0.048). It was also found that the expression rate of ARC was correlated with the age of the AML patients, where younger patients had elevated levels of ARC as compared to that of the older patients (*P* =0.05). In terms of the clinical parameters, there was an association between ARC expression levels and the percentage of bone marrow blasts (*P* =0.046), whereas the percentage of the bone marrow blasts decreased as the percentage of promyelocytes increased, with a concomitant upregulation of ARC. Regarding the expression rate of ARC in different AML subtypes, AML-M3 was found to exhibit the highest expression rates of ARC with a median RQ value of 14.23 folds (data not shown). In addition, there was a negative correlation between ARC expression rates and the FLT3-mutation status (*P* =0.047), where AML patients with normal wildtype FLT3 genotype showed higher expression rates of ARC (mean value=34.4 folds) as compared to that of those who had FLT3 mutations (5.72 folds). Also, it was found that the expression of ARC was positively correlated with the expression of CD117 surface marker which is a marker for poor prognosis. ARC expression rate was also positively correlated with ASXL1 expression (*P* <0.001) (Figure 3B) and PHF6 expression (*P<0.001*) (Figure 3C).

BAX expression

**Figure 4 -:**
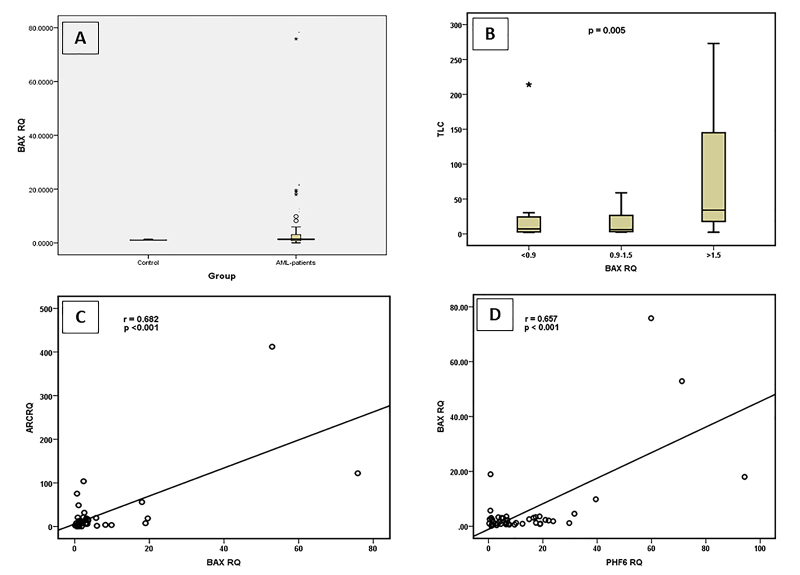
Significance of BAX expression. BAX expression levels, represented as RQ value, in AML patients and in Control groups (A). Correlation between BAX expression and TLC (B). Correlation between BAX expression and ARC expression (C). Correlation between BAX expression and PHF6 expression (D).

**Figure 5 -:**
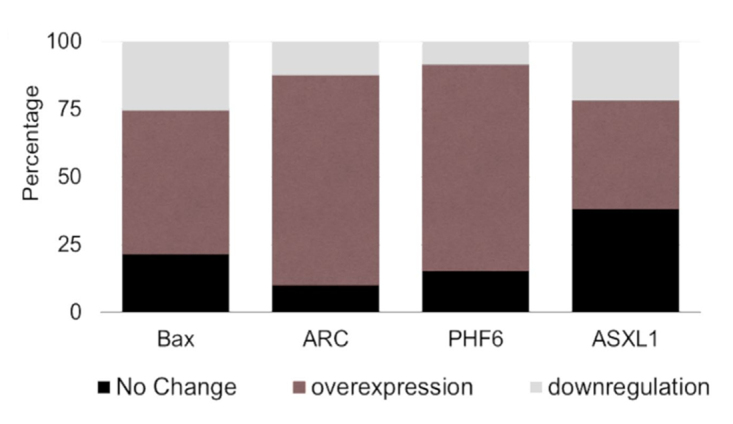
Distribution of markers in AML patients. Graphical representation of the expression profiling of the selected markers showing the percentage of patients that showed no change or exhibited overexpression or downregulation.

In the case of the pro-apoptotic marker, the expression levels of BAX in AML patients was not significantly different from normal subjects where 55.56% of the AML patients showed elevated expression rates of this marker (Figure 4A and Figure 5). Consistent with the expression pattern of ARC, BAX had the lowest expression rates in AML-M3 subtype (data not shown). Regarding the co-expression of BAX with myeloid cell surface markers in AML patients, there was a highly significant negative correlation between BAX expression rates and CD7 (*P*=0.01). BAX was also found to be positively correlated with TLC (*P*=0.005) (Figure 4B). In addition, ARC expression rates were correlated with BAX expression levels (Figure 4C) and PHF6 expression levels (Figure 4D).

Cumulatively, the RQ data of the selected genes *ARC*, *PHF6*, *ASXL1* and *BAX*, revealed that there was a highly significant correlations between ARC expression levels and that of PHF6 (r =0.545; **P** <0.001) as well as with that of ASXL1 (r =0.652; *P* <0.001). Similarly, BAX expression rates showed highly significant correlation with that of ARC (r =0.682; *P* <0.001), and PHF6 (r =0.657; *P*<0.001), while a slight positive correlation was found with ASXL1 (r =0.488; *P* =0.001).

Survival analysis

**Figure 6 -:**
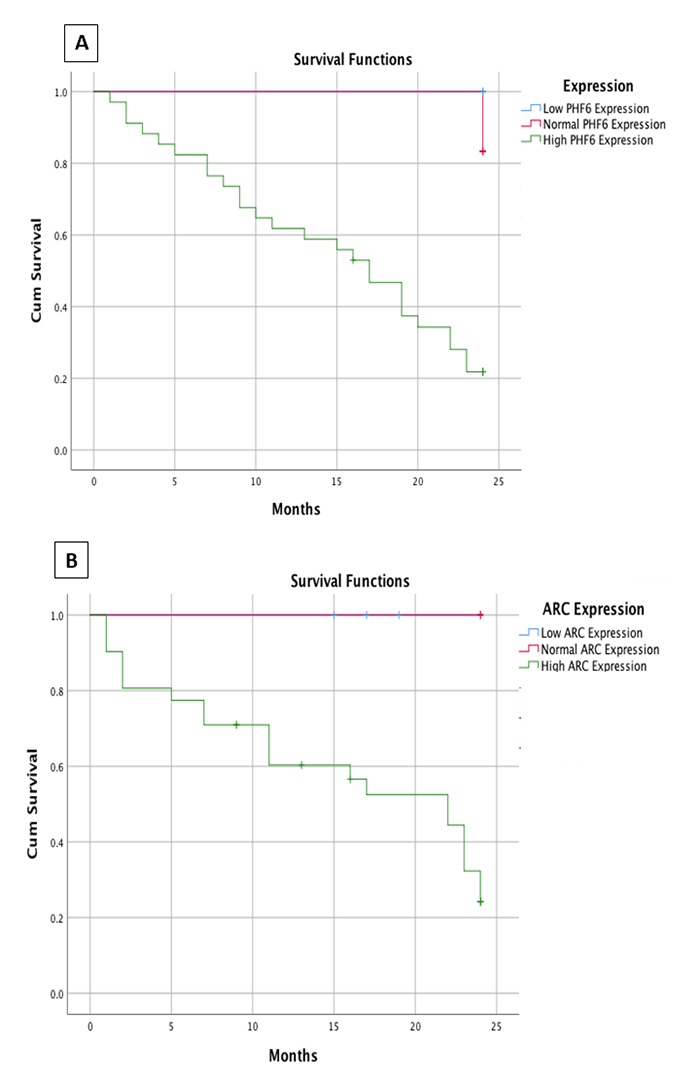
Survival analysis in AML patients. Kaplan-Meier survival analysis of overall survival (A) according to PHF6 expression levels and (B) according to ARC expression levels. The Plots showed that AML patients with high PHF6 or ARC transcript levels have lower MST than patients with normal or low expression levels (log-rank *P* value is 0.02 and 0.026 respectively).

To assess the prognostic potential of ARC and PHF6, Kaplan-Meier survival analysis was performed to study the correlation between the levels of *PHF6* and *ARC* gene expression and overall survival for 2 years (Figure 6). AML patients were divided into 3 groups based on the expression levels of the tested markers as compared to that of the control group (upregulation, no change and down regulation). 

In case of PHF6, the number of patients with high PHF6 expression levels is n= 43 (the number of the censored cases in this group is n=2), and the number patients with normal PHF6 expression levels is n= 3 (the number of the censored cases in this group is n=1), and the number of patients with low PHF6 expression levels is n= 8 (the number of the censored cases in this group is n=1). The total number of cases is n= 54, of whom 4 cases counted as censored.

We found that cases with low PHF6 expression levels had significantly longer overall survival than those with high expression levels where the median survival time (MST) in the patients with low expression levels were 2 years (only one patient died after 2 years and the rest of the patients survived until the end of the study period), however the mean survival time for the patients with high expression levels was about 6 months from the date of diagnosis (*P*= 0.02 ) (Figure 6A).

On the other hand, ARC gene showed an upregulation in 45 patients (n=45), 4 of whom were counted as censored (n=4), and the number of patients with normal ARC expression levels is n=4, and the number of patients with ARC low expression levels is n= 5. The total number of cases is n= 54, of whom 4 cases were counted as censored.

In addition, cases with low ARC expression levels had significantly longer overall survival than those with high ARC expression levels where the median survival time (MST) in the patients with low and normal expression levels were 2 years (survived until the end of the study period), however, the MST of patients with high expression was 10 months (*P*= 0.026 ) (Figure 6B). 

In addition, in multivariate analysis, PHF6 high expression was found to be correlated with poor OS (Hazard Ratio 3.6, *P*= 0.032). Also, ARC high expression was correlated with poor OS (Hazard Ratio 2.8, *P*= 0.021) and hence it indicates poor therapeutic response.

## Discussion

Modulations of gene expression that result from initiation and progression of any deviation of cellular contexts from normal scenarios is considered one of the most reliable determinants for monitoring pathological processes. 

In the present study, the same path was followed in an attempt to correlate the levels of the selected genes’ expression patterns with the pathogenesis of adult AML. Therefore, specific genes were selected, namely *ASXL1*, *BAX*, *NOL3* (*ARC*) and *PHF6*, which represent key points in vital cellular pathways and are thought to play roles in the onset and progression of AML. Thus, may help to shed light on the biology of Leukemic stem cells and to delineate the associated molecular mechanisms. 

Plant homeodomain (PHD)-like family (PHF) member 6 (*PHF6*), which contains two PHD zinc-finger domains *PHF6*, was our second target gene. Our data revealed an over-expression trend of *PHF6* represented by a marked increase in its transcripts levels observed in 77.8% of the patients. This up-regulation of *PHF6* gene in AML is in agreement with the study of Landais *et al*. (2005), who found a *PHF6* over-expression in the *Kis2*-rearranged tumors, providing the first evidence to link disruption of *PHF6* and the progression of hematologic malignancy. Similarly, an up-regulation of *PHF6* gene in cases of lymph node metastasis of breast cancer was correlated with tumor proliferation (Yu *et al*., 2012). In addition, ALL1, a human homologue of *Drosophila* trithorax protein, which contains PHD zinc-finger domain, as well as other genes expressing proteins with PHD zinc-finger domains are also known to be frequently mutated in ALL and AML (Gu *et al*., 1992; Tkachuk *et al*., 1992; Nakamura *et al*., 1993; Parry *et al*., 1993; Chesi *et al*., 1998).

*PHF6* expression was found to play a role in the determination of AML prognosis, since the high expression levels was correlated with poor clinical outcome and low survival time. In agreement to our study, the study of Hajjari *et al.* (2016) revealed increased or exclusive expression of the mutant PHF6 in acute leukemia in addition it was remarkably overexpressed in many cancer types such as breast and colorectal cancers and can function as oncogenic factor in several types of cancer 

*NOL3,* or *ARC*, also followed the same expression pattern where, the expression of *ARC* gene was significantly increased among AML patients where 83.3% of the patients were expressing higher levels of the ARC gene more than that of the individuals of the healthy groups. Similar to our data, a study by Wang *et al*. (2005) revealed that ARC was over-expressed in human cancer cells, where the quantified protein by western blot was over-expressed in nine out of ten cancer cell lines representing pancreatic /colorectal /breast /lung /lymphoma /cervical /prostate /glioblastoma carcinoma. Similarly*,* Carter *et al*. (2011) evaluated ARC protein expression in AML patients and tried to correlate expression levels with clinical picture. They found that ARC expression was variable among the AML samples. It is worth mentioning that the authors found that ARC levels have a prognostic meaning since higher levels were associated with low overall survival.

Furthermore, the data also revealed that ARC expression was significantly correlated with clinical characteristics. In term of clinical classification, ARC’s transcript levels were higher in FAB types M4 and M3 and lower in type M2, when compared to healthy individuals. ARC expression was also variable among patients with different genetic translocations and was not associated with cytogenetic abnormalities. In addition, elevated ARC levels were observed in patients with *t*(15;17) translocation. On the other hand, ARC levels were significantly lower in patients with mutated NPM1, with either wild-type or mutated FLT-3, which means that ARC levels do not depend on the status of FTL3 mutation. Similarly, ARC levels showed significantly negative correlation with peripheral blood blast but not the bone marrow blasts. In addition, no significant relationship was found between ARC level and patient status or WBCs/platelet count or Hb levels. Similary ARC levels were not found associated with cytogenetic groups or with FLT-3 mutation status (Carter *et al*., 2011).

ARC protein functions as anti-apoptotic factor and many studies conducted on ARC concluded that it is a strong independent adverse prognostic marker in AML. Moreover, ARC was found to augment resistance to chemotherapeutic agents in AML and the decrease in ARC levels sensitize AML cells to chemotherapy since ARC increases chemokine CCL2, CCL4, and CXCL12 expression in mesenchymal stromal cells (MSC) and facilitates leukemia-microenvironment interactions (Carter *et al*., 2016). Our results are in agreement with such studies since the analysis revealed that patients with high ARC expression levels had low OS than patients with normal or low ARC levels.

## Conclusion

The cumulative analysis performed for the data obtained in this study highlighted the significance of the assessment of the expression profiles for the selected markers. The positive correlations found among these markers in AML cases, especially between ARC and the other markers, in particular with PHF6, point out to the diagnostic potential of these two markers in surveying cases with hematopoietic disturbances. Furthermore, when correlating such findings with patients’ outcomes, it was revealed that ARC and PHF6 have great potential and can be considered of prognostic value to predict treatment responsiveness and hence may lead to reaching a consensus recommendation that would help in case management and patients’ stratification.

## Supplementary Material

The following online material is available for this article:

Table S1 - Clinical data of AML patients.

Table S2 - Frequency of occurrence of cell surface markers of AML patients.
