# Supplementary material for: Expression profiling of some Acute Myeloid Leukemia - associated markers to assess their diagnostic / prognostic potential
Source: Genet Mol Biol. 2021 Jan 6;44(1):e20190268. doi: 10.1590/1678-4685-GMB-2019-0268 (PMC7802071; doi:10.1590/1678-4685-GMB-2019-0268)
Supplement: Table S1 - [file 1415-4757-GMB-44-1-e20190268-s1.pdf]

Genetics and Molecular Biology

https://doi.org/10.1590/1678-4685-GMB-2019-0268

Supplementary Material to "Expression Profiling of Some Acute Myeloid Leukemia - Associated Markers to Assess their Diagnostic / Prognostic Potential"

Table S1 - Clinical data of AML patient group. Corresponding values of control subjects were within normal range (data not shown).

Patient #	Age	Gender	FAB Classification	Hemoglobin	Platelets	TLC	BA%
1	50	Female	AML-M4	7.2	44	181.27	82%
2	25	Female	AML-M4	6.5	27	145.09	90
3	42	Female	AML-M3	9.9	22	2.87	4
4	49	Female	AML-M2	4.4	11	34	27
5	58	Female	RAEB-MDS-II (AML on top of MDS)	7	30	2.2	15
6	25	Male	AML-M1	10	173	18.3	95
7	40	female	AML-M1	3	79	7.23	71
8	21	female	AML-M2	6.4	27	4.32	70
9	52	female	AML-M4				36
10	59	Male	AML-M1	7.4	14	3.8	86
11	21	Male	AML-M2 (on top of CML)	7.3	17	9.95	54
12	68	female	AML-M1	7.5	29	26.5	79
13	68	female	AML-M0	10.4	77.9	30.2	93
14	51	Male	AML-M3	10	25	2.7	4
15	45	female	AML-M4	9.2	25	14	30
16	27	female		6.2	241	273	
17	30	Male	AML-M1	8.4	36	22	79
18	46	Male	AML-M1	5.2	28	33.31	93
19	26	female	AML-M1	6.9	10	129.89	92
20	48	Male	AML-M2	2.9	6	6	48
21	46	Male	AML-M1	8	42	214	76
22	54	Male	AML-M5b	9.4	26	63.5	38
23	41	Male	AML-M3	10	23	3	15
24	25	Male	AML-M2	7.4	382	3.4	18
25	45	Male	AML-M0	10.2	78	29.8	90
26	21	Male	AML-M3	9.4	22	2.9	6
27	40	Male	AML-M1	8.5	8	75	74
28	49	female	AML-M4	7.3	43	179.5	79%
29	26	female	AML-M4	6.4	26	144.3	89
30	43	female	AML-M3	10	21	3.01	5
31	48	female	AML-M2	4.4	10.8	35	28
32	57	female	RAEB-MDS-II (AML on top of MDS)	6.9	29	2.3	16
33	26	Male	AML-M1	9.9	172.5	17.9	94
34	39	female	AML-M1	3.1	77	7.18	72
35	22	female	AML-M2	5.9	29	4.5	70
36	49	female	AML-M4	8	50	169	36
37	57	Male	AML-M1	7.4	14	3.8	86
38	21	Male	AML-M2 (on top of CML)	7.3	17	9.95	54
39	68	female	AML-M1	7.5	29	26.5	79
40	68	female	AML-M0	9.9	77.9	30.2	93
41	51	Male	AML-M3	10	25	2.7	4

Patient #	Age	Gender	FAB Classification	Hemoglobin	Platelets	TLC	BA%
42	44	female	AML-M4	8.9	23.5	15.5	30
43	28	female	AML-M1	6.5	250	267	
44	30	Male	AML-M1	8.1	35.9	24	79
45	46	Male	AML-M1	5.2	28	33.31	93
46	26	female	AML-M1	7.1	9.5	131	92
47	48	Male	AML-M2	2.9	5.7	6.2	48
48	46	Male	AML-M1	7.5	36	213	76
49	54	Male	AML-M5b	9.1	25	59	38
50	41	Male	AML-M3	10	23	2.9	15
51	25	Male	AML-M2	7.2	400	3.2	18
52	45	Male	AML-M0	10.2	78	29.8	90
53	21	Male	AML-M3	9.2	21	2.7	6
54	39	Male	AML-M1	8.7	7	81	74
