# Supplementary material for: Expression profiling of some Acute Myeloid Leukemia - associated markers to assess their diagnostic / prognostic potential
Source: Genet Mol Biol. 2021 Jan 6;44(1):e20190268. doi: 10.1590/1678-4685-GMB-2019-0268 (PMC7802071; doi:10.1590/1678-4685-GMB-2019-0268)
Supplement: Table S2 - [file 1415-4757-GMB-44-1-e20190268-s2.pdf]

Genetics and Molecular Biology

https://doi.org/10.1590/1678-4685-GMB-2019-0268

Supplementary Material to "Expression Profiling of Some Acute Myeloid Leukemia - Associated Markers to Assess their Diagnostic / Prognostic Potential"

Table S2 - Frequency of occurrence of cell surface markers of AML patients.

Cell surface marker	Frequency of occurrence	
	Number (n=54)	Percentage
MPO	54	100
CD13	54	100
CD33	50	92.6
CD4	9	16.6
CD11c	10	18.5
CD117	20	37.0
MHC-II	32	59.3
CD14	6	11.1
Tdt	2	3.7
CD34	25	46.3
CD7	8	14.8
